# Testing the validity and reliability of the Chinese version of the Staden schizophrenia anxiety rating scale

**DOI:** 10.3389/fpsyt.2022.992745

**Published:** 2022-09-20

**Authors:** Chenyuan Du, Jiayue Chen, Xiaoyan Ma, Wenzhen Tu, Langlang Chen, Jian Liu, Dan Zhou, Xinying Chen, Jiulong Zhang, Hongjun Tian, Chuanjun Zhuo, Deguo Jiang

**Affiliations:** ^1^Department of Psychiatry, Wenzhou Seventh Peoples Hospital, Wenzhou, China; ^2^Department of Psychiatry, Nankai University Affiliated Tianjin Fourth Center Hospital, Tianjin Fourth Center Hospital, Tianjin, China; ^3^Department of Psychiatry, Nankai University Affiliated Tianjin Anding Hospital, Tianjin Medical University Clinical Hospital of Mental Health, Tianjin Anding Hospital, Tianjin, China; ^4^Department of Neuroimagine, Nankai University Affiliated of Tianjin Anding Hospital, Tianjin Medical University Clinical Hospital of Mental Health, Tianjin Anding Hospital, Tianjin, China; ^5^Department of Nurse Management, Nankai University Affiliated Tianjin Anding Hospital, Tianjin Medical University Clinical Hospital of Mental Health, Tianjin Anding Hospital, Tianjin, China

**Keywords:** S-SARS, Chinese version, reliability, validity, anxiety, schizophrenia

## Abstract

Accurate assessment of anxiety disorders and their symptomatology in schizophrenic patients is important for prognosis and treatment. Measuring anxiety on the traditional anxiety assessment scales such as the Hamilton Anxiety Rating (HAMA) Scale or the self-rating depression scale (SAS) is challenging and often considered unsuitable for assessing anxiety symptoms in patients with schizophrenia. The Staden schizophrenia anxiety rating scale (S-SARS) has been shown to reliably measure specified and undifferentiated anxiety in schizophrenia. The present study aims to test the reliability and validity of the S-SARS version, thereby facilitating Chinese psychiatrists in assessing anxiety symptoms in schizophrenic patients. A total of 300 patients meeting ICD-10 diagnostic criteria of schizophrenia were recruited by convenience sampling. We used the exploratory factor analysis (EFA) to evaluate the structural validity of S-SARS and receiver operating characteristic (ROC) curves to acquire the cutoff point of S-SARS to define the severity of anxiety. Internal consistency was assessed using Cronbach's and Krippendorff's α scores. 1-week test-retest reliability was assessed using the intra-class correlation coefficient (ICC). Correlation analysis with HAMA was used to determine the Chinese version of S-SARS criterion validity. We have the following results: Our version of S-SARS showed Cronbach's α score as 0.899, Krippendorff's α as 0.874, and a correlation coefficient of 0.852 between S-SARS and HAMA. The EPA demonstrated that the contribution rate of major factors was 69.45%. All the items of S-SARS were located in one factor and showed a high factor load (0.415–0.837). The correlation coefficient of S-SARS and HAMA was 0.852. Our results indicated that Chinese version of S-SARS showed good constructive validity and reliability. It also showed better criterion validity compared to HAMA. The S-SARS and its Chinese version can thus serve as an effective tool for assessing anxiety symptoms in patients with schizophrenia.

## Introduction

Anxiety is one of the prominent features observed among patients with schizophrenia. It often precedes and accompanies schizophrenia and is one of its risk factors (1–5). A meta-analysis of 52 studies showed that the prevalence rate of anxiety and related disorders in schizophrenia is 38.3% (2), and a recent study reported it to be higher, 45% (4). The anxiety symptoms observed in schizophrenic patients have characteristics similar to the general anxiety disorder and some distinct (6–9). The established diagnostic anxiety syndromes in schizophrenia are panic disorder, social anxiety disorder, specific phobias, obsessive-compulsive disorder, post-traumatic stress disorder, and generalized anxiety disorder. The simultaneous presence of symptoms related to specific anxiety disorders and undifferentiated anxiety in schizophrenic patients negatively impacts the course and prognosis of this disorder (10–12). Typical clinical features differentiate patients with schizophrenia anxiety from patients experiencing general anxiety (9, 13–17). Patients with schizophrenia anxiety express hyper-vigilance, restlessness, trembling, palpitations, and tension. However, these symptoms do not compete with the diagnostic criteria of any of the specified anxiety syndromes such as generalized anxiety, panic episode, and social phobia; hence, these symptoms are also defined as un-differentiated anxiety symptoms (17–21). In contrast, differentiated anxiety symptoms are usually demonstrated under anxiety spectrum disorders with mainly identified specific fears, panic attacks, obsessions, compulsions, and excessive worries (22).

Unspecific anxiety symptoms are often confused as psychotic symptoms of hyper-phobia that are subsequently confused with the tense state caused by auditory verbal hallucinations (23). General and systematic reviews on the existing scales developed to measure levels of anxiety in schizophrenic patients are inadequate and non-specific. The Hamilton Anxiety Rating Scale (HAMA) is one of the first rating scales used to measure the severity of perceived anxiety symptoms. It is one of the most widely used anxiety rating scale, however, this scale has been reported to have several drawbacks. It has been suggested to capture symptoms thought to be characteristic of depression but not anxiety and also does not adequately measure worry, a key feature of anxiety. Smith et al. in their review of 17 anxiety measuring scales from 11 studies found them inadequate against standardized quality assessment criteria, and no single measure of anxiety demonstrated strong psychometric properties or adequate methodological quality. They recommend the BAI (Beck Anxiety Index), DASS (Depression Anxiety Stress Scale), or SAES (Scale of Anxiety Evaluation in Schizophrenia) for general screening, and the DGSS (DSM-based Generalized Anxiety Disorder Symptoms Severity), LSAS (Liebowitz Social Anxiety Scale), OCI (Obsessive–Compulsive Inventory), PSI (the Psychological Stress Index), PTQ (Perseverative Thinking Questionnaire), and Y-BOCS (Yale-Brown Obsessive Compulsive Scale) to assess symptoms associated with specific anxiety disorders. Of these the Scale of Anxiety Evaluation in Schizophrenia (SAES) was designed for the schizophrenia population. Its conceptual scope is broad and includes items like decreolization, indecision, and pain, that are arguably expressions of anxiety, but it excludes compulsions. In addition, all its items are taken from existing anxiety scales, and does not account specifically for anxiety that is expressed within delusional content and in disturbances of perceptions (24). The underwhelming psychometric properties of the standard anxiety scales in the schizophrenia population may be an expression of clinical complexity in this population. Anxiety is rather difficult to assess during an acute phase of schizophrenia owing to the psychotic symptoms characteristic of this phase (18). Further, anxiety may clinically be difficult to distinguish from akathisia, which is a common extrapyramidal side effect of antipsychotic medication (25). Compounding this complexity further, psychotic features and akathisia may exacerbate anxiety and vice versa (18, 25). Concurrent comorbid depressive features also complicate assessments as depressive features correlate with anxiety both in the general (20) and schizophrenia populations (21). To address these shortcomings, the following objectives for measuring anxiety validly and reliably in schizophrenia are inferred: It should account for the anxiety that is expressed within delusional content and in disturbances of perceptions, and also should be discerned from the features of schizophrenia. Moreover, it should account for both the various specified kinds of anxiety as well as undifferentiated anxiety.

Hence, Van Staden et al. developed a scale named Staden Schizophrenia Anxiety Rating Scale (S-SARS) (26), which had 3 sub-scales, including 18 items, and was explicitly used to assess the quantity of anxiety symptoms in schizophrenia patients. Each item has six narrative anchor points on a scale from 0 to 5 to indicate the severity of the anxiety during the preceding week and was accompanied by pre-designed guided questions during the interview to inform the ratings. The first part is used to assess the specific anxiety, and the first item of this sub scale is “no persecutory or nihilistic anxiety during the past 7 days,” the second item is “unclear whether persecutory or nihilistic anxiety has been present or absent, ” the third item is “The patient has been concerned about the attitude, intentions, or plans of other beings toward him/her, or the patient is afraid that life has lost its meaning for him/her, ” the fourth item is “The patient is afraid that he/she may be persecuted, or may be a victim of malicious intent, or the patient is afraid that his/her life or livelihood is drawing to an end,” the fifth item is “The patient is afraid as part of his/her belief that something bad or harmful is about to be done to him/her, or the patient is afraid as part of his/her belief that his/her life is in danger.,” the sixth item is “The patient is afraid as part of his/her belief that something bad or harmful is being done or was done to him/her, or the patient is afraid as part of his/her belief that he/she is dying, decaying, or ceasing to exist fully.” The second subscale of S-SARS consists of six items, the first item is “No anxiety attacks during the past 7 days,” the second item is “Unclear whether anxiety attacks have been present or absent,” the third item is “The patient has been concerned about things or people he/she hears, sees, feels tactually, smells or tastes, and these sensory perceptions are dreams, images, illusions, or hallucinations”, the fourth item is “The patient has been afraid of the things or people he/she hears, sees, feels tactually, smells or tastes, and these sensory perceptions are dreams, images, illusions, or hallucinations.”, the firth item is “The patient has been afraid and has been startled by the things or people he/she hears, sees, feels tactually, smells or tastes, and these sensory perceptions are dreams, images, illusions, or hallucinations.”, the sixth item is “The patient has been afraid or scared when objectively hallucinating.”. The third subscale also included 6 items, the first item is “No anxiety attacks during the past 7 days,” the second item is “Unclear whether anxiety attacks have been present or absent,” the third item is “The patient has had at least one discreet episode of intense fear without much concern about a further attack or its cause or its implications,” the fourth item is “The patient has had at least one discreet episode of intense fear, and has been concerned about a further episode or its cause or its implications,” the fifth item is “The patient has had more than one discreet episode of intense fear and has been very concerned about a further episode or its cause or its implications,” the sixth item is “The patient has had more than one discreet episode of intense fear, and has been very concerned that these episodes may be an indication of his/her impending death (26).

The three subscales of S-SARS all had the guidelines, such as “Consider all information pertaining to delusions, if present, for potential relevance,” and “Consider all information pertaining to self-worth and appraisal of own resources (livelihood) for potential relevance,” “Consider all information pertaining to dreams, images, illusions & hallucinations, if present,” and “Consider information about any discreet episodes of intense fear, even if not a “panic attack” in the strict sense, and even if the cause of the fear is known.” Simultaneously, all the three subscales had Minimum enquiries to assure the scales could be used accurately. After the S-SARS was used in clinical practices, its psychometric properties were observed to be different. Hence, the developer of S-SARS analyzed the data from these studies, pooled it separately, and reported its validity and reliability in acute and residual phases of schizophrenia. After pooled analysis, the data demonstrated a better psychometric property. They approved its validity and reliability for measuring the specified and undifferentiated anxiety in schizophrenia patients, providing an accurate measurement of anxiolytic treatment effects (1). Considering this as a reference, we tried to introduce S-SARS use in Chinese patients to protect the prognostic of the patients better.

## Materials and methods

### Material

Patients presenting to Wenzhou Seventh Peoples' Hospital from July 2020 to July 2021 and meeting ICD-10 diagnostic criteria for schizophrenia were recruited by convenience sampling. A total of 300 patients were enrolled. Duration of schizophrenia ranged from 6 to 28 months, with an average of 11.0 ± 2.5 months and with an average of 28.45 ± 4.30 years. To consider the acute phase diagnosis, the score requirement should be 60 or more through the Structured Clinical Interview for the Positive and Negative Syndrome Scale (PANSS) (27). Participants willing to be in the study should sign the informed consent form and are affirmed in an informed consent document. The ethics committee of Tianjin Fourth Center Hospital approved this study. Regarding the exclusion criteria, if they self-report or document the clinical condition as severe physical diseases history, they were excluded from the study, followed by loss of consciousness, unstable or significant medical disorders, past head injury with a neurological sequel, or intellectual disability.

### Methods

The S-SARS was performed by categorizing the data into two major phases: (1) translated to the Chinese version, and (2) Psychometric evaluation of the reliability and validity of the Chinese version.

#### Step 1: Translated to the Chinese version

S-SARS is a free-charge scale that can be freely acquired from the related network in English and Chinese versions. For better understanding purposes, the English version of the S-SARS was translated into a Chinese version. Step-wise instructions were followed: First: To perform forward translation, two senior professional psychiatrists from the department of psychological medicine, The Tianjin Fourth Center Hospital, who knew speaking native Chinese and were familiar with English, conducted the forward translation and generated the Chinese version of S-SARS.

#### Step 2: Back translation

An independent expert with a doctoral degree and native of English speaking also familiar with the Chinese language performed back translations. Soon after completing the back translations, they formed two English versions, i.e., one is the original S-SARS and the back-translated S-SARS). A harmonization meeting was conducted by two translators who detected the inconsistency among different versions of translations. If discrepancies were identified, then further clarification was expected from the developer to ensure the conceptual validity of all the translated versions. After the completion of harmonization, the final version of the S-SARS was completed.

### Reliability and validity of the Chinese version of the S-SARS evaluation

The present study was approved by the ethical committee of Tianjin Fourth Center Hospital. The psychometric properties of S-SARS included internal consistency, reliability, and test-retest reliability constructive validity. In this study, 12 senior psychiatrists independently performed the psychoanalysis of 300 patients using the Chinese version of S-SARS. The data was not shared between the evaluators. When all the data was acquired, Cronbach αand Krippendorff's α were calculated to evaluate the reliability of the Chinese version of S-SARS. Krippendorff's αwas used to test the inter-rater reliability and Cronbach αco-eficient was used to measure the scale reliability (15). The exploratory factor analysis (EPA) was applied to assess the constructive validity of the S-SARS (28). Correlation with HAMA was used to calculate the criterion validity.

### Cutoff point acquired

In the present study, according to the clinical doctor's definition, the receiver operating characteristic (ROC) graph curve (29) was analyzed to define the cutoff point of the severity degree of the anxiety symptoms.

## Results

### Reliability

The Cronbach's α of the Chinese version of S-SARS was 0.899, and Krippendorff's alpha of the Chinese version of S-SARS was 0.874; therefore, these data supported the Chinese version of S-SARS for conferring good reliability.

### Validity

Through factor analysis, our data demonstrated the major factors' contribution rate, which was 69.45%. In addition, all the items of S-SARS located in one factor have a high factor load between 0.515 and 0.837. Hence, this data supports the Chinese version of S-SARS for having good constructive validity, the detailed information listed in the Table 1. The correlation coefficient of S-SARS and HAMA was 0.852; this data also suggested that S-SARS has better criterion validity.

**Table 1:** Factor loading display.

**Items**	**Factor loading**	**95% confidence interval**
1. No persecutory or nihilistic anxiety during the past 7 days	0.415	0.337–0.680
2. Unclear whether persecutory or nihilistic anxiety has been present or absent	0.567	0.488–0.701
3. The patient has been concerned about the attitude, intentions, or plans of other beings toward him/her, or the patient is afraid that life has lost its meaning for him/her	0.485	0.411–0.542
4. The patient is afraid that he/she may be persecuted, or may be a victim of malicious intent, or the patient is afraid that his/her life or livelihood is drawing to an end	0.511	0.477–0.630
5. The patient is afraid as part of his/her belief that something bad or harmful is about to be done to him/her, or the patient is afraid as part of his/her belief that his/her life is in danger	0.798	0.655–0.906
6. The patient is afraid as part of his/her belief that something bad or harmful is being done or was done to him/her, or the patient is afraid as part of his/her belief that he/she is dying, decaying, or ceasing to exist fully.	0.793	0.700–0.824
7. No anxiety attacks during the past 7 days	0.813	0.733–0.900
8. Unclear whether anxiety attacks have been present or absent	0.637	0.598~0.700
9. The patient has been concerned about things or people he/she hears, sees, feels tactually, smells or tastes, and these sensory perceptions are dreams, images, illusions, or hallucinations	0.552	0.478–0.699
10. The patient has been afraid of the things or people he/she hears, sees, feels tactually, smells or tastes, and these sensory perceptions are dreams, images, illusions, or hallucinations	0.425	0.397–0.598
11. The patient has been afraid and has been startled by the things or people he/she hears, sees, feels tactually, smells or tastes, and these sensory perceptions are dreams, images, illusions, or hallucinations	0.489	0.407–0.536
12. The patient has been afraid or scared when objectively hallucinating.	0.837	0.759–0.903
13. No anxiety attacks during the past 7 days	0.455	0.405–0.500
14. Unclear whether anxiety attacks have been present or absent	0.753	0.711–0.799
15. The patient has had at least one discreet episode of intense fear without much concern about a further attack or its cause or its implications	0.704	0.639–0.897
16. The patient has had at least one discreet episode of intense fear, and has been concerned about a further episode or its cause or its implications	0.587	0.513–0.636
17. The patient has had more than one discreet episode of intense fear and has been very concerned about a further episode or its cause or its implications	0.636	0.578–0.877
18. The patient has had more than one discreet episode of intense fear, and has been very concerned that these episodes may be an indication of his/her impending death	0.700	0.544–0.811

### Cutoff point

Experienced by ROC, our data demonstrated that the S-SARS score was ≥6 mild severity anxiety syndrome with good sensitivity and specificity of 0.942 and 0.759, respectively, and an AUC of 0.676. If S-SARS is ≥ 12, schizophrenia patients should suffer from severe anxiety syndrome, and the sensitivity and specificity were found to be 0.919 and 0.855, respectively, with 0.799 AUC (29).

## Discussion

Till now, recent review findings have found that there is no apparatus to measure anxiety accurately in the Chinese patients with schizophrenia. However, the present study was conducted to check the reliability and validity of the Chinese version of S-SARS. The reliability evaluation results showed that S-SARS had a high consistency among different assessments from different doctors and had a good internal consistency of the scale. The evaluation results demonstrated that the S-SARS scale's structural validity was also ideal. ROC curve showed that when the scale score was ≥6, the patients usually suffered from mild severity of S-SARS within the last week. If the S-SARS score is ≥12, the patients should show the symptoms of severe S-SARS within the last week (30, 31). More notably, the correlation analysis with HAMA showed that S-SARS could be used as an index to assess the anxiety and severity in schizophrenic patients.

In this study, the sensitivity and specificity were considered, and it was suggested that the standard for clinical use should be ≥6 points. 45.7% of workers were observed to have mild anxiety severity within the last week. When the threshold for clinical use is stable by 12 points, 25.4% of the patients were observed to have severe anxiety severity within the last week; when the S-SARS range was between 6 to12, 28.7% of workers had moderate anxiety severity. The score of S-SARS positively correlated with the HAMA scores, *r* = 0.891, *P* < 0.05. Therefore, when used in further clinical and research practice, S-SARS properties might improve the accurate measurement of anxiety in schizophrenia patients. The S-SARS measures both the specified anxiety disorders and also undifferentiated anxiety disorders. However, it merely measures one of the specified anxiety disorders. Several beneficial reasons exist while measuring anxiety accurately in schizophrenia patients; it improves and clarifies various clinical and research reasons. When such anxiety is overlooked, it increases the schizophrenia burden in a patient by conferring a negative impact on the quality of life (18), functioning (19, 25), overall psychopathology, and the severity of comorbid medical conditions (20).

In the clinical practices, anxiety in schizophrenia may compound the morbidity and mortality similar to that of syndromal anxiety. Syndromal anxiety negatively impacts the quality of life (18), functioning (25), overall psychopathology and the severity of comorbid medical conditions (20). Increased rates of relapse, more frequent and longer duration of hospitalizations, poorer response to pharmacological treatments, substance abuse, negative attribution style, suicide and suicide attempts has been associated with anxiety in schizophrenia (21). In addition, anxiety is difficult to recognize during an acute phase of schizophrenia owing to the psychotic symptoms of this phase (12, 21). Further, anxiety may be clinically difficult to distinguish from the common extrapyramidal side effect of anti-psychotic medication, akathisia (7). Again, psychotic features and akathisia may exacerbate anxiety and vice versa (12, 21). Similarly, comorbid depressive features correlate with anxiety both in the general (8) and schizophrenia populations (9). Considering these complexities in acute-phase schizophrenia (14), more studies need to be conducted on the role of psychotic symptoms, akathisia and depressive features in verifying whether undifferentiated anxiety is empirically discernible from syndromal anxiety and no anxiety. Verifying undifferentiated anxiety in schizophrenic patients would warrant further research into its prevalence, contribution to morbidity, etiology and treatment.

## Conclusions

The above data showed that the S-SARS has proved to be a good prognostic marker in evaluating the reliability and validity and has better sensitivity and specificity to assess the severity of anxiety in schizophrenic patients within the last week. Therefore, the Chinese version of S-SARS can be applied to measure the anxiety severity within schizophrenic patients within the last week, to provide useful information to give tailored treatment to these patients as early as possible by improving the prognostic features of these patients.

## Data availability statement

The original contributions presented in the study are included in the article/supplementary materials, further inquiries can be directed to the corresponding authors.

## Ethics statement

The studies involving human participants were reviewed and approved by Tianjin Fourth Center Hospital. The patients/participants provided their written informed consent to participate in this study.

## Author contributions

CD, JC, and HT: conceptualization, methodology, analysis, software, investigation, and writing/original draft preparation. WT, LC, and JL: software, analysis, writing/reviewing, and editing. DZ, XC, and JZ: software, investigation, writing/reviewing, and editing. XM, DJ, and CZ: conceptualization and supervision. All authors contributed to the article and approved the submitted version.

## Funding

This work was sponsored by grants from the National Natural Science Foundation of China (81871052 and 82171503), the Key Projects of the Natural Science Foundation of Tianjin (17JCZDJC35700), the Tianjin Health Bureau Foundation (2014KR02), and the Tianjin Science and Technology Bureau (15JCYBJC50800).

## Conflict of interest

The authors declare that the research was conducted in the absence of any commercial or financial relationships that could be construed as a potential conflict of interest.

## Publisher's note

All claims expressed in this article are solely those of the authors and do not necessarily represent those of their affiliated organizations, or those of the publisher, the editors and the reviewers. Any product that may be evaluated in this article, or claim that may be made by its manufacturer, is not guaranteed or endorsed by the publisher.

## References

[1] Naidu K van Staden W Fletcher L. Discerning undifferentiated anxiety from syndromal anxiety in acute-phase schizophrenia. Ann Gen Psychiatry. (2020) 19:26. 10.1186/s12991-020-00277-4 32318113 PMC7158120

[2] Achim AM Maziade M Raymond É Olivier D Mérette C Roy MA. How prevalent are anxiety disorders in schizophrenia? A meta-analysis and critical review on a significant association. Schizophr Bull. (2011) 37:811–21. 10.1093/schbul/sbp148 19959704 PMC3122284

[3] Nebioglu M Altindag A. The prevalence of comorbid anxiety disorders in outpatients with schizophrenia. Int J Psychiatry Clin Pract. (2009) 13:312–7. 10.3109/13651500903094559 24916943

[4] Kiran C Chaudhury S. Prevalence of comorbid anxiety disorders in schizophrenia. Ind Psychiatry J. (2016) 25:35–40. 10.4103/0972-6748.196045 28163406 PMC5248417

[5] Seedat S Fritelli V Oosthuizen P Emsley RA Stein DJ. Measuring anxiety in patients with schizophrenia. J Nerv Ment Dis. (2007) 195:320–4. 10.1097/01.nmd.0000253782.47140.ac 17435482

[6] Bosanac P Mancuso SG Castle DJ. Anxiety symptoms in psychotic disorders: results from the second Australian national mental health survey. Clin Schizophr Relat Psychoses. (2016) 10:93–100. 10.3371/1935-1232-10.2.93 27440210

[7] Van Putten T. The many faces of akathisia. Compr Psychiatry. (1975) 16:43–7. 10.1016/0010-440x(75)90019-x 234073

[8] Howland RH Rush AJ Wisniewski SR Trivedi MH Warden D Fava M. Concurrent anxiety and substance use disorders among outpatients with major depression: Clinical features and effect on treatment outcome. Drug Alcohol Depend. (2008) 99:248–60. 10.1016/j.drugalcdep.08010 18986774

[9] Fusar-Poli P Nelson B Valmaggia L Yung AR McGuire PK. Comorbid depressive and anxiety disorders in 509 individuals with an at-risk mental state: Impact on psychopathology and transition to psychosis. Schizophr Bull. (2014) 40:120–31. 10.1093/schbul/sbs136 23180756 PMC3885287

[10] Smith EL Garety PA Harding H Hardy A. Are there reliable and valid measures of anxiety for people with psychosis? A systematic review of psychometric properties. Psychol Psychother Theory Res Pract. (2021) 94:173–98. 10.1111/papt.12265 31880406

[11] Naidu K van Staden WC van der Linde M. Severity of psychotic episodes in predicting concurrent depressive and anxiety features in acute phase schizophrenia. BMC Psychiatry. (2014) 14:166. 10.1186/1471-244X-14-166 24903304 PMC4068766

[12] Temmingh H Stein DJ. Anxiety in patients with schizophrenia: Epidemiology and management. CNS Drugs. (2015) 29:819–32. 10.1007/s40263-015-0282-7 26482261

[13] Majadas S Olivares J Galan J Diez T. Prevalence of depression and its relationship with other clinical characteristics in a sample of patients with stable schizophrenia. Compr Psychiatry. (2011) 53:145–51. 10.1016/j.comppsych.03009 21621754

[14] Veras AB Cougo S Meira F Peixoto C Barros JA Nardi AE. Schizophrenia dissection by five anxiety and depressive subtype comorbidities: Clinical implications and evolutionary perspective. Psychiatry Res. (2017) 257:172–8. 10.1016/j.psychres.07048 28763736

[15] Dlagnekova A van Staden W Masenge A. Validity and reliability of the Vigour Assessment Scale in avolitional schizophrenia outpatients. Schizophr Res. (2021) 235:36–43. 10.1016/j.schres.07018 34304145

[16] Kay SR Opler LA Lindenmayer JP. Reliability and validity of the positive and negative syndrome scale for schizophrenics. Psychiatry Res. (1988) 23:99–110. 10.1016/0165-1781(88)90038-8 3363019

[17] Liemburg E Castelein S Stewart R van der Gaag M Aleman A Knegtering H. Genetic Risk and Outcome of Psychosis(GROUP) Investigators Two subdomains of negative symptoms in psychotic disorders: Established and confirmed in two large cohorts. J Psychiatr Res. (2013) 47:718–25. 10.1016/j.jpsychires.01024 23472837

[18] Priebe S McCabe R Junghan U Kallert T Ruggeri M Slade M. Association between symptoms and quality of life in patients with schizophrenia: a pooled analysis of changes over time. Schizophr Res. (2011) 133:17–21. 10.1016/j.schres.09021 22001495

[19] Romm KL Melle I Thoresen C Andreassen OA Rossberg JI. Severe social anxiety in early psychosis is associated with poor premorbid functioning, depression, and reduced quality of life. Comp Psychiatry. (2012) 53:434–40. 10.1016/j.comppsych.06002 21821242

[20] Wigman JT van Nierop M Vollebergh WA Lieb R Beesdo-Baum K Wittchen HU. Evidence that psychotic symptoms are prevalent in disorders of anxiety and depression, impacting on illness onset, risk, and severity—implications for diagnosis and ultra–high risk research. Schizophr Bull. (2012) 38:247–57. 10.1093/schbul/sbr196 22258882 PMC3283146

[21] Tarrier N Picken A. Co-morbid PTSD and suicidality in individuals with schizophrenia and substance and alcohol abuse. Soc Psychiatry Psychiatr Epidemiol. (2011) 46:1079–86. 10.1007/s00127-010-0277-0 20711764

[22] Garay RP Samalin L Hameg A Llorca PM. Investigational drugs for anxiety in patients with schizophrenia. Expert Opin Investig Drugs. (2015) 24:507–17. 10.1517/13543784.2014.987339 25423603

[23] Jouini L Ouali U Ouanes S Djebara MB Nacef F Gouider R. Akathisia among patients undergoing antipsychotic therapy: prevalence, associated factors, and psychiatric impact. Clin Neuropharmacol. (2022) 10:506. 10.1097./WNF.0000000000000506 35696611

[24] Kessler RC Demler O Frank RG Olfson M Pincus HA Walters EE. Prevalence and treatment of mental disorders, 1990 to 2003. N Engl J Med. (2005) 352:2515–23. 10.1056/NEJMsa043266 15958807 PMC2847367

[25] Achim AM Ouellet R Lavoie MA Vallières C Jackson PL Roy MA. Impact of social anxiety on social cognition and functioning in patients with recent-onset schizophrenia spectrum disorders. Schizophr Res. (2013) 145:75–81. 10.1016/j.schres.01012 23415473

[26] Van Staden W Dlagnekova A Naidu K. Validity and reliability of the Staden schizophrenia anxiety rating scale. Diagnostics. (2022) 12:831. 10.3390/diagnostics12040831 35453879 PMC9028449

[27] Kay SR Opler LA Spitzer RL Williams JB Fiszbein A Gorelick A. SCID-PANSS: two-tier diagnostic system for psychotic disorders. Compr Psychiatry. (1991) 32:355–61. 10.1016/0010-440x(91)90085-q 1935026

[28] Yockey RD Kralowec CJ. Confirmatory factor analysis of the procrastination assessment scale for students. Sage Open5. (2015) 2158244015611456. 10.1177/2158244015611456

[29] Mvududu NH Sink CA. Factor analysis in counseling research and practice. Couns Outcome Res Eval. (2013) 4:75–98. 10.1177/2150137813494766

[30] Hasanagic A Ozsagir CB. The validity exploration of general procrastination scale (Lay, 1986). Epihany. (2020) 11:55. 10.21533/epiphany.v11i1.283

[31] Ferrari JR. Reliability of academic and dispositional measures of procrastination. Psychol Rep. (1989) 64:1057–8. 10.2466/pr0.643c.1057

